# Fetal cardiac masses

**DOI:** 10.1007/s00247-025-06431-y

**Published:** 2025-10-22

**Authors:** Bhagyashree Rathore, Marissa E. Adamson, Lara E. Berklite, Pradipta Debnath, Beth M. Kline-Fath, Kimberley G. Miles, Cara E. Morin, Kristin A. Schneider

**Affiliations:** 1 Cincinnati Children’s Hospital Medical Center, 3333 Burnet Ave, Cincinnati, OH 45229 USA; 2 University of Cincinnati Medical Center, Cincinnati, USA

**Keywords:** Fetus, Fibroma, Hemangioma, Heart neoplasms, Rhabdomyoma, Teratoma

## Abstract

Although rare in the pediatric population, cardiac tumors represent a significant portion of prenatally detected masses, second only to sacrococcygeal teratomas. Fetal cardiac masses are predominantly benign, with rhabdomyoma being the most common fetal cardiac mass in the setting of tuberous sclerosis. Multiple lesions are pathognomonic for rhabdomyomas and prompt evaluation for tuberous sclerosis. Fibroma, teratoma, and hemangioma are other consistently detected cardiac tumors. In this article, we review the epidemiology, imaging, treatment, and outcomes of these fetal cardiac masses, emphasizing the role of imaging in surveillance, identifying complications, refining the differential diagnosis, and recognizing potential diagnostic pitfalls.

**Graphical abstract:**

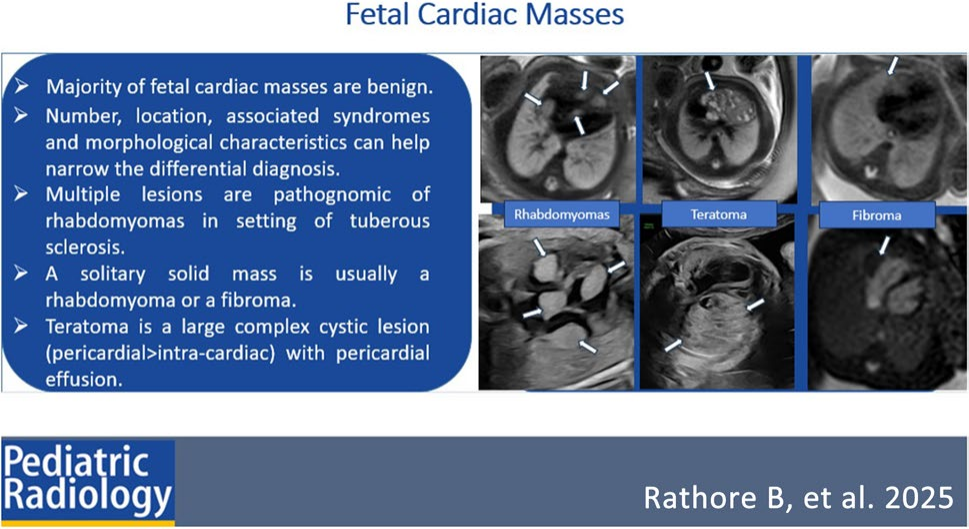

## Introduction

Congenital tumors comprise approximately 2% of pediatric masses, with fetal cardiac masses representing up to 22% of all cases [1]. The reported incidence of fetal cardiac masses ranges from 0.009% to 0.2%, and with advances in fetal imaging and increased use of fetal echocardiography and magnetic resonance imaging (MRI), cardiac lesions are increasingly detected prenatally [2]. Most prenatally detected cardiac masses are benign neoplasms, with rare case reports of malignant lesions such as rhabdomyosarcoma and fibrosarcoma [3–5]. The most common fetal cardiac tumor is rhabdomyoma in the setting of tuberous sclerosis complex (TSC). Syndromes such as Gorlin-Goltz syndrome, Carney complex, and Beckwith-Wiedemann syndrome have an overall increased incidence of cardiac tumors later in life; however, cardiac lesions associated with these syndromes are not usually detected prenatally [6–8]. Given the location of the masses and risks to the mother and fetus, it is typically not possible to obtain tissue for histopathology prenatally. As a result, non-invasive imaging plays an important role in the diagnosis, surveillance, and management of fetal cardiac masses.

This review explores the evaluation of fetal cardiac masses, emphasizing the role of imaging in surveillance, identifying complications, and refining the differential diagnosis, while recognizing potential diagnostic pitfalls.

## Fetal cardiac imaging: overview and imaging algorithms

Ultrasound is the primary imaging modality for evaluating the fetus, both structurally and functionally. If a cardiac mass is identified on antenatal second-trimester anatomic ultrasound, patients are usually referred to a fetal program for further evaluation with fetal echocardiography and often fetal MRI [9]. Assessment by fetal echocardiography is typically sufficient for antenatal guidance and postnatal planning; however, whenever possible, a fetal MRI is performed. Fetal MRI provides additional high soft tissue definition and morphological assessment, allowing for better evaluation of the extent and number of masses [10]. It also provides an excellent evaluation of the entire fetus and is especially useful to assess for anomalies in other organ systems that can be seen with genetic syndromes [10–12].

### Fetal echocardiography

Fetal echocardiography is the preferred initial imaging modality for the diagnosis and surveillance of fetal cardiac masses due to the greater availability, ease of performance, and increased patient comfort. Fetal echocardiography should document the characteristics of the mass (e.g., location, echogenicity) as well as cardiac function, rhythm, and blood flow patterns to understand the hemodynamic impact of the lesion. Echocardiographic characteristics of the mass, including solid versus cystic nature, echogenicity, vascularity, and calcifications, also help to narrow the differential diagnosis. While the location of the cardiac mass can aid in narrowing the differential diagnosis, the extent of the mass(es) can be difficult to ascertain by fetal echocardiography, particularly if the mass is large and distorts the normal anatomic relationships.

Fetal echocardiography plays an important role in assessing the hemodynamic impact of cardiac masses. Depending on mass location, blood flow into or out of the heart can be impacted. Estimation of the cardiac output by fetal echocardiography can aid in identifying hemodynamic compromise [13]. Evaluation of the ductus venosus, umbilical artery, and vein is also important in the assessment of overall fetal well-being, particularly if there is concern that the mass is affecting fetal blood flow [13, 14]. Pericardial effusion, ascites, pleural effusions, and skin edema can also be identified by fetal echocardiography. The presence of hydrops is an ominous sign in any fetus, and in those with cardiac masses, careful interrogation is needed to assess for cardiac compromise.

#### Technique

In fetal echocardiography, a combination of standard views and sweeps from multiple views, along with color and spectral Doppler, should be used to assess mass location and its impact on surrounding structures (Table 2). A 4-chamber view with the heart oriented vertically (equivalent to an apical 4-chamber view on transthoracic echocardiography) can be used to assess atrio-ventricular valve inflow and regurgitation. The left ventricular outflow tract is often well visualized from a 4-chamber horizontal view, but ideal spectral Doppler interrogation is from a 4-chamber vertical view swept cranially toward the outflow. Continuing to sweep cranially from the horizontal view, the aortic and ductal arches can be seen and interrogated with color and spectral Doppler. The right ventricular outflow tract and pulmonary valve can be assessed from the horizontal or sagittal view, as can the superior and inferior vena cava. Ventricular function can be quantitatively and qualitatively assessed by standard metrics on fetal echocardiography, including myocardial performance index (Tei) and ventricular shortening fractions [11]. A proposed fetal echocardiography protocol is summarized in Table 1.

**Table 1:**
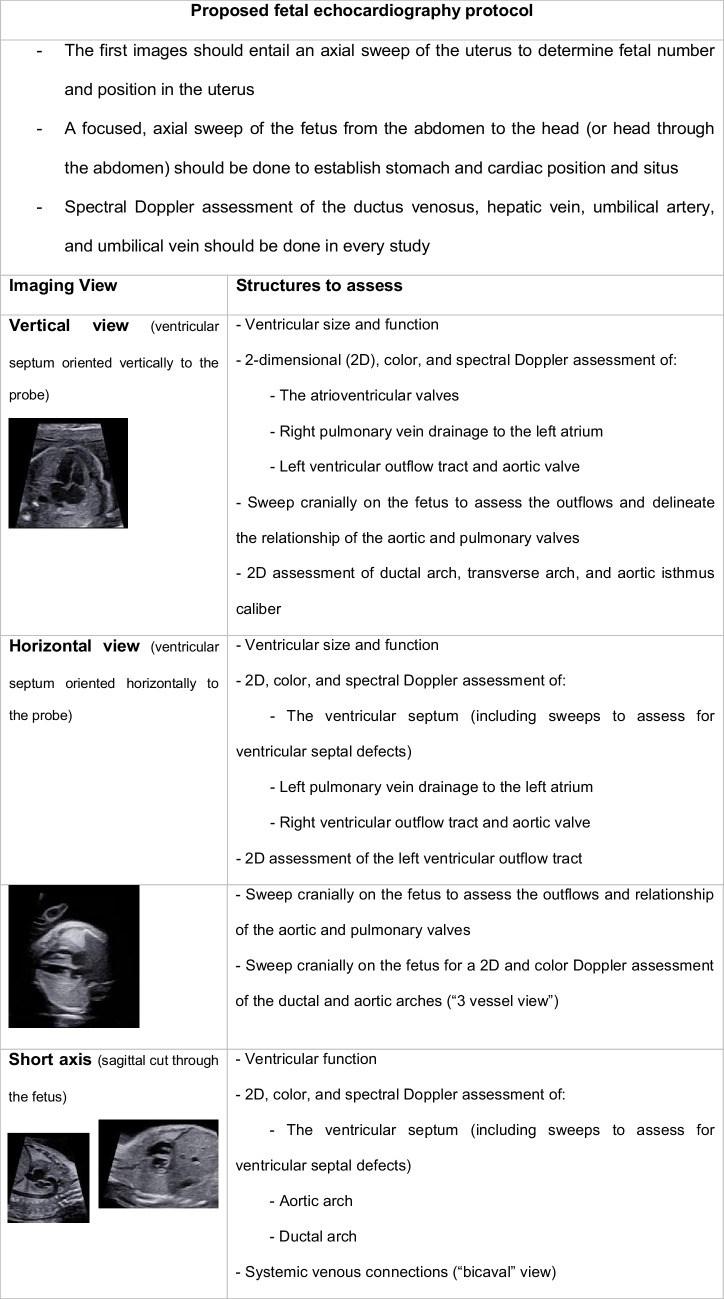
Summary of proposed fetal echocardiography protocol along with relevant imaging views

### Fetal MRI

Fetal MRI has proven to be an effective technique in the evaluation of the fetus. Images are obtained with single-shot rapid acquisition, which limits fetal motion artifacts, offering excellent soft tissue detail and a large field of view that cannot be achieved with routine sonography or echocardiography. There is currently no evidence that MR imaging is harmful to the fetus at 1.5 or 3 tesla. Acoustic damage from loud tapping, teratogenic effects, and elevated SAR during fetal imaging, even with the use of 3-T scanners, have not been proven [12, 15–17]. Gadolinium administration is contraindicated, as it has been shown to increase the risk of harm to the fetus [21]. Known limitations include sequences with less signal than conventional imaging, artifacts from fetal and maternal motion, MR artifacts especially at 3 T, and the inability to image due to maternal contraindications or claustrophobia.

Given these advantages, fetal cardiac MRI is often performed in the presence of a fetal cardiac mass to offer additional assessment of tissue composition and superior visualization of extra-cardiac structures. It allows for the evaluation of multiple masses and provides detailed information about tissue characteristics, including signal intensity, location, and extent of the mass, and assessment of mass effect on regional structures. Fetal MRI can also identify signs of fetal hydrops and other extra-cardiac abnormalities that may suggest underlying genetic conditions, such as subependymal nodules associated with tuberous sclerosis complex (TSC).

Fetal functional and advanced cardiac MRI is an emerging technique that uses Doppler-enabled gating of the fetal heart rate to produce dynamic and motion-corrected images of the heart [18]. Primarily performed in fetuses with cardiac disease between 30–40 weeks of gestation, bright blood gated cine imaging allows for functional information such as blood flow, cardiac physiology, and chamber volumes [19, 20].

Motion-corrected slice-to-volume registration tools facilitate the reconstruction of images from motion-corrupted MRI stacks. Initially implemented to generate isotropic images of the fetal brain, these tools have now been expanded to create three-dimensional reconstructions of fetal heart anatomy [25]. Thin single-shot fast spin-echo (SSFSE) or half-Fourier single-shot turbo spin-echo (HASTE) images can be reconstructed using slice volume reconstruction (SVR) algorithms to create motion-corrected two-dimensional images for interpretation, which can be processed to create three-dimensional images [21, 22]. These three-dimensional reconstructions have been shown to improve visualization of fetal heart and cardiovascular anatomy, particularly in the third trimester [23].

Fetal MRI is likely to become an increasingly valuable complementary tool for evaluating the hemodynamic and functional impact of cardiac masses on cardiac structures. However, further research is needed to determine its utility and effectiveness at earlier gestational ages [23].

#### Technique

Rapid acquisition sequences are preferred to mitigate fetal and maternal motion (Table 2). Fetal MRI protocols typically include assessment of the maternal uterus and the entire fetus, including dedicated brain and body imaging as well as optional fetal cardiac gated sequences. The most common static sequences are balanced steady state free precession (bSSFP), which produces bright blood images, and single-shot fast spin-echo (SSFSE) or half-Fourier single-shot turbo spin-echo (HASTE), which produce dark-blood images. These provide anatomical evaluation and tissue characterization of the mass and its relationship to cardiovascular and other mediastinal structures. T1 images can allow for the assessment of blood products or fat in some cases. Diffusion-weighted imaging is typically reserved for brain and renal lesions. Gadolinium-based contrast agents are contraindicated in fetal MRI. A proposed fetal MRI protocol is summarized in Table 2.

**Table 2:** Summary of a proposed fetal MRI protocol

Body part	Planes	Sequences	Important structures to assess
**Uterus**	Axial, coronal, and sagittal	Large field of view balanced steady state free precession (bSSFP) to include whole uterus, 5 mm	Uterine myometrium for fibroids, C-section scar, adnexa, placenta, visualized maternal structures (e.g. - kidneys for cysts/angiomyolipoma, spine)
	Sagittal	T2 single-shot turbo spin-echo (SSH TSE)	Cervix
**Fetal body**	Axial, coronal, and sagittal	Single-shot fast spin-echo (SS FSE)	Any additional congenital abnormalities, especially fetal kidneys in suspected cases of tuberous sclerosis, hydrops
	Axial, coronal, and sagittal	Balanced steady state free precession (bSSFP)	
	Coronal, sagittal	Fast spoiled gradient echo (FSPGR)	
**Fetal brain**	Axial, coronal, sagittal	Single-shot fast spin-echo (SS FSE)	Structural abnormalities
	Axial	Diffusion-weighted imaging (DWI)	Infarct/ischemia
	Axial	Echo planar imaging (EPI) blood	Intracranial hemorrhage
**Fetal cardiac – cine**	Axial 4 chamber	Balanced turbo field echo (BTFE) through heart	In evaluation of cardiac mass(es) - the location, number, extent, mass effect on adjacent structures
	2 chamber		
	Short axis		
**Fetal cardiac - static**	Axial, coronal, sagittal	1.25 mm single-shot black blood (SSH BB)	
	Oblique tilt 15° from straight axial		
	Oblique tilt 15° sagittal with aorta		

## Imaging in cardiac tumor genetic predisposition syndromes

Genetic testing plays a significant role in the evaluation of fetuses with cardiac masses. Patients with certain genetic syndromes, such as tuberous sclerosis complex, Gorlin-Goltz syndrome, and Carney complex, are at a higher risk of developing cardiac tumors (Table 3). Fetal echocardiography is the mainstay of imaging surveillance in these cases. Many syndromes do not have specific surveillance recommendations or society-level guidelines; however, where possible, we have included reasonable surveillance recommendations. If a cardiac mass is identified, the frequency of follow-up is dictated by the mass location and the physiological impact on cardiac output and rhythm.

**Table 3:** Summary of genetic predisposition syndromes and associated cardiac tumors

Syndrome	Associated gene	Cardiac tumor	Extra-cardiac findings	Reference
Tuberous sclerosis complex	TSC1, TSC2	Rhabdomyoma	**Brain**: subependymal nodules, subependymal giant cell astrocytoma, cortical/subcortical tubers, hemimegaloencephaly **Renal**: cysts – both macrocysts and microcysts have been reported Angiomyolipoma (prenatal detection is uncommon but has been reported in 33-week fetus)	[24, 25]
Basal cell nevus syndrome/Gorlin-Goltz syndrome	PTCH1, SUFU	Cardiac fibroma	Fetal ocular anomalies including microphthalmia and vertebral segmentation anomalies have been reported The head and neck findings of odontogenic keratocysts, dural calcification, and medulloblastoma are not reported antenatally	[26]
Carney complex	PRKAR1A	Myxoma	Typically, all known findings of Carney complex, including mucosal myxoma, melanotic schwannoma, testicular tumors manifest in adulthood with no consistent reported fetal anomalies. Published case reports of maternal Carney complex in pregnancy describe intra-uterine growth restriction and adrenal insufficiency in neonates	[27, 28]

## Fetal cardiac masses

The most common fetal cardiac mass is rhabdomyoma, followed by teratoma and fibroma (Table 4) [29]. Other masses reported include hemangioma, myxoma, pericardial cyst, and lipoma. While myxomas comprise about 20% of pediatric cardiac tumors, these are extremely uncommon prenatally, with less than 50 cases described in the literature [29]. Malignant masses such as fibrosarcoma and rhabdomyosarcoma are exceedingly rare, with only a few published case reports [5, 30].

Prenatal imaging-based diagnosis of cardiac masses can be challenging due to overlapping features. Intra-cardiac versus pericardiac nature of the lesions can be difficult to ascertain prenatally. However, a few important diagnostic clues, like multiplicity, location, echogenicity, and associated extra-cardiac anomalies, can help narrow the differential diagnosis. We review the epidemiology, imaging features, outcomes, and management of commonly encountered fetal cardiac masses.

### Rhabdomyoma

#### Epidemiology

Rhabdomyoma is the most common fetal and neonatal cardiac neoplasm accounting for 60–85% of fetal cardiac tumors [2, 31]. There is a strong association with tuberous sclerosis complex (TSC) [29, 32, 33]. Rhabdomyomas associated with TSC can be seen in the second trimester [34, 35]. *TSC 2* gene mutations are more common than *TSC 1* in fetuses with cardiac rhabdomyomas [36]. Multiple lesions are observed in 80–83% of fetuses with TSC while a solitary lesion is seen in 16–19% cases [37]. Cardiac rhabdomyoma without TSC is rare but has been reported [38].

Rhabdomyomas are most commonly located in the ventricular myocardium but can also occur in the atria or on the epicardial surface [39]. The left ventricle is the most common location [36, 40]. Most lesions are sessile; however, pedunculated lesions have been described [32]. Lesions protruding into the cardiac chambers can cause hemodynamic compromise. Rarely, large lesions causing inflow or outflow obstruction can lead to fetal hydrops. Significant cardiac arrhythmias have been described in 16% of patients, particularly if the atrio-ventricular groove is involved [41].

Tumor growth is often biphasic with lesions increasing until approximately 32 weeks of gestation, at which point enlargement typically slows and lesions can even regress in size [39, 42].

#### Echocardiographic features

Rhabdomyomas are typically homogeneous, hyperechoic, well-circumscribed, often multiple masses within the myocardium. Most rhabdomyomas are asymptomatic, but careful interrogation for inflow or outflow obstruction is necessary to determine if changes in delivery planning are needed due to mass effect causing hemodynamically important obstruction. This is typically done by documenting laminar color flow across the atrio-ventricular and semilunar valves and looking for valve regurgitation. Flow in the aortic and ductal arches should be assessed on every fetal echocardiogram and should be antegrade in both arches. Reversal of flow in either arch can indicate significant outflow tract obstruction, requiring the initiation of prostaglandin therapy after birth to maintain adequate pulmonary or systemic blood flow. Fetal heart rate and rhythm should also be assessed as difficult-to-manage fetal arrhythmias could alter delivery planning.

#### MRI features

**Fig. 1:**
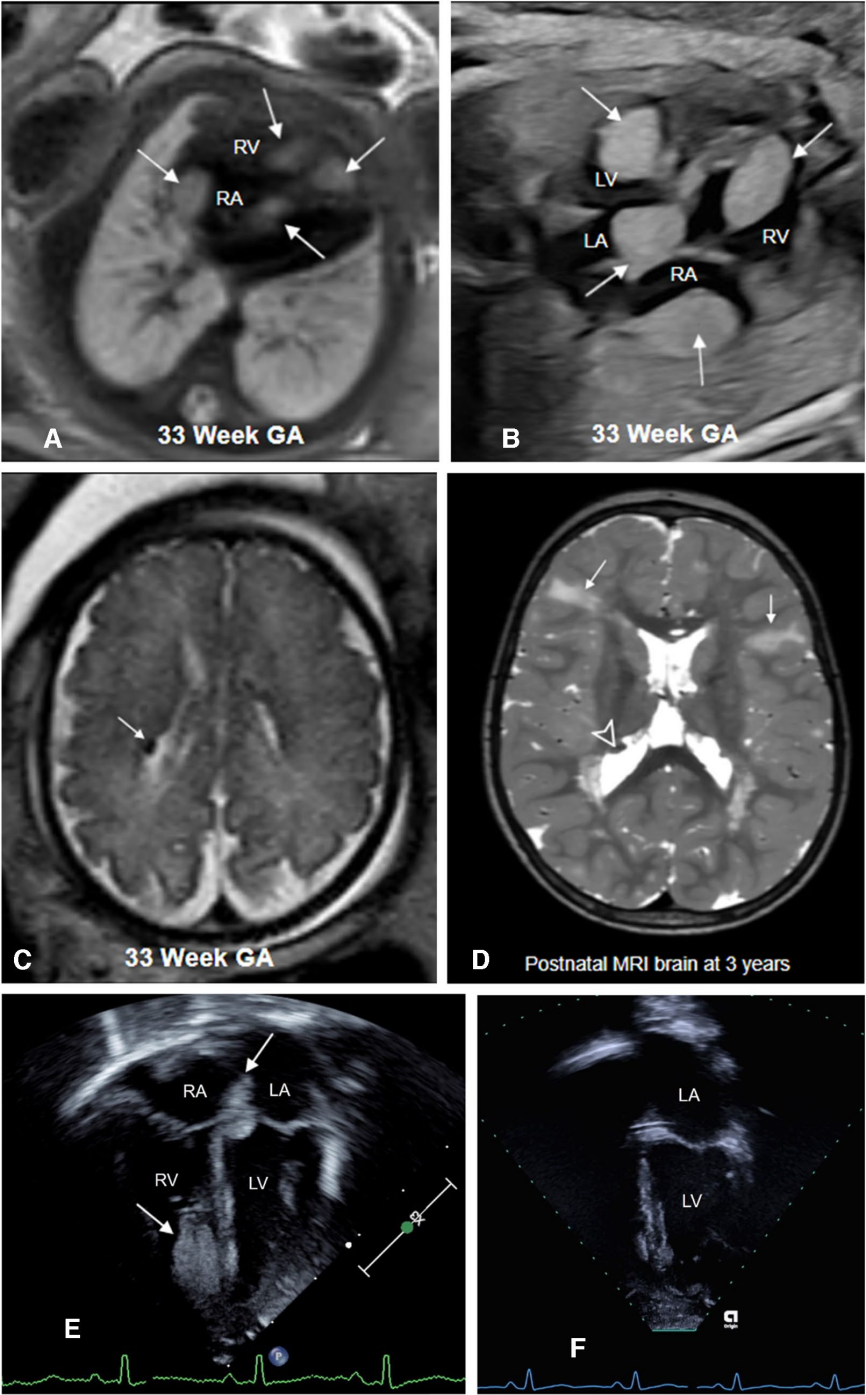
A 33-week gestational age (GA) fetus with confirmed TSC2 gene mutation. **A** Axial T2-weighted image shows multiple T2 hyperintense ovoid lesions (*arrows*) involving the myocardium and inter-ventricular septum. **B** Four-chamber echocardiography image demonstrating multiple ovoid hyperechoic masses (*arrows*) in the same location as the MR. **C** Axial balanced steady state free precession (bSSFP) brain image showing a hypointense subependymal nodule (*arrow*) along the right lateral ventricle. The patient received mTOR inhibitors. **D** Axial T2-weighted image of the brain at 3 years of age showing multiple cortical, subcortical lesions/tubers (*arrows*), and subependymal nodules (*arrowhead*) typical of tuberous sclerosis. Postnatal echocardiogram in an apical 4-chamber view confirmed the presence of rhabdomyomas (*arrow*) in the same locations identified prenatally (**E**), but over time they reduced in size, with the most recent echocardiogram performed at 5 years of age showing no intra-cardiac tumors (**F**). *LV*, left ventricle; *RV*, right ventricle; *LA*, left atrium; RA, right atrium

**Fig. 2:**
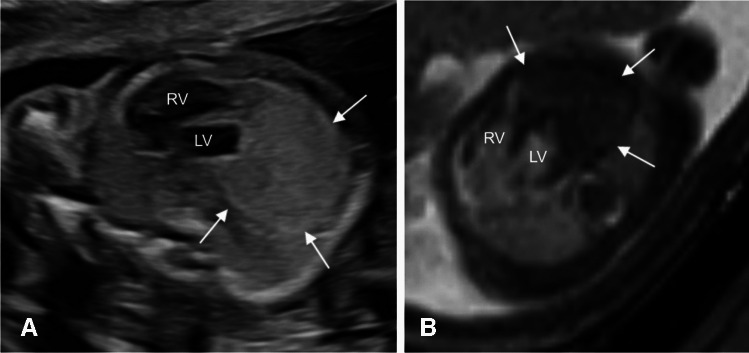
A fetal cardiac mass detected in a 20-week gestational age pregnancy. **A** Horizontal view echocardiogram demonstrating a large, well-defined hyperechoic mass at the cardiac apex (*arrows*). **B** Axial T2-weighted image shows a large solitary T2 hypointense mass (*arrows*). No additional lesions were seen. Imaging of fetal brain and kidneys was unremarkable. The differential considerations were fibroma or rhabdomyoma. Unfortunately, the pregnancy resulted in fetal demise, with genetic testing positive for tuberous sclerosis and an autopsy confirming a rhabdomyoma. *LV*, left ventricle; *R* right ventricle

On MRI, rhabdomyomas are homogeneous, T1 isointense to hyperintense, and T2 hyperintense relative to myocardium [43]. The masses usually do not demonstrate cystic changes or calcifications. The extent of larger lesions can be better delineated on MRI. A thorough review of the fetal brain and kidneys can demonstrate additional abnormalities related to tuberous sclerosis. Fetal MRI in the second trimester can demonstrate subependymal nodules, cortical/subcortical lesions, subependymal giant cell astrocytoma, megalencephaly, or hemi-megalencephaly [25, 44]. Renal cysts – solitary or multiple – can be seen in fetuses with tuberous sclerosis. Case reports of fetuses with tuberous sclerosis and polycystic kidney disease have been documented and are attributed to the close proximity of these genes on chromosome 16 [45]. Renal angiomyolipoma is rarely detected prenatally but has been reported [24]. Figures 1 and 2 show cardiac rhabdomyomas in fetuses with tuberous sclerosis.

#### Treatment and outcomes

Most fetal and neonatal rhabdomyomas are asymptomatic and regress by 4 years of age [46]. Once fetal rhabdomyomas are detected, dedicated fetal echocardiography is performed to detect individuals with a high risk of hemodynamic compromise. In our experience, at least one imaging review is recommended in the third trimester. Maternal sirolimus is emerging as a promising treatment for symptomatic fetal rhabdomyomas, with several case reports and literature reviews supporting its efficacy [42, 47, 48]. All patients who received intra-uterine treatment were monitored with frequent echocardiography to assess the treatment response [47, 48].

For children with asymptomatic rhabdomyomas, echocardiography every few years is recommended until regression of the rhabdomyomas is documented [49, 50]. In children with larger masses affecting valve function or causing outflow tract obstruction, the cadence of surveillance echocardiography is guided by severity. In children with tuberous sclerosis, medical management with off-label use of mTOR inhibitors (sirolimus and everolimus) can be used to treat arrhythmias and has been associated with more rapid tumor regression [42, 47]. Though rare, surgical indications for resection include the presence of hemodynamically significant outflow tract obstruction, valve dysfunction, or intractable arrhythmias [51].

### Teratoma

#### Epidemiology

Fetal teratomas are rare tumors, with an incidence of one in 40,000 live births [52]. These can occur in the midline, anywhere from the coccyx to the pineal [53]. In a study by Simonini et al. the most common locations for fetal teratomas were in the sacrococcygeal region (59.5%), neck (20.2%), and oropharynx (7.6%), while pericardial teratomas account for only 0.04% of reported cases. Although rare, teratomas are the second most common fetal cardiac tumors, accounting for 9.5% to 19% of fetal cardiac masses [54].

Teratomas can arise from the ventricular or atrial myocardium, but they are most commonly pericardial rather than intra-myocardial. Teratomas are typically solitary complex cystic lesions and are frequently associated with a pericardial effusion [29, 55]. Rapid tumor growth can be seen between 20 weeks and 40 weeks of gestation with resultant mass effect on the heart and great vessels [56]. Pericardial teratomas are more frequently located on the right side of the heart and typically attached to the great vessels via a pedicle [57]. Among reported cases of intra-cardiac teratomas, the inter-ventricular septum is the most common location with the mass projecting into the right ventricle more often than the left [58]. Prenatally, cardiac/pericardial teratomas can sometimes be difficult to distinguish from other mediastinal masses, such as a large mediastinal teratoma. However, identification of a normal thymus and right paramedian location favors a pericardiac origin [55].

#### Echocardiographic features

Teratomas are typically large, well-circumscribed, heterogeneous masses. A pericardial effusion is commonly seen [59]. In addition to characterizing the location and imaging characteristics of the mass, fetal echocardiography should assess for mass effect, such as obstruction to systemic venous return or flow through the great vessels. There is a potential for rapid growth of these masses, leading to compression of critical structures and fetal demise or hydrops prenatally [60]. If present, the size and location of a pericardial effusion should also be noted. Echocardiographic findings typical of cardiac tamponade are not seen on fetal echocardiography, but the cardiac function, inflow, and outflow can be impacted. Doppler assessment of the hepatic veins and ductus venosus can identify right-sided compression from an effusion or mass effect, which may have clinically significant postnatal implications.

#### MRI features

**Fig. 3:**
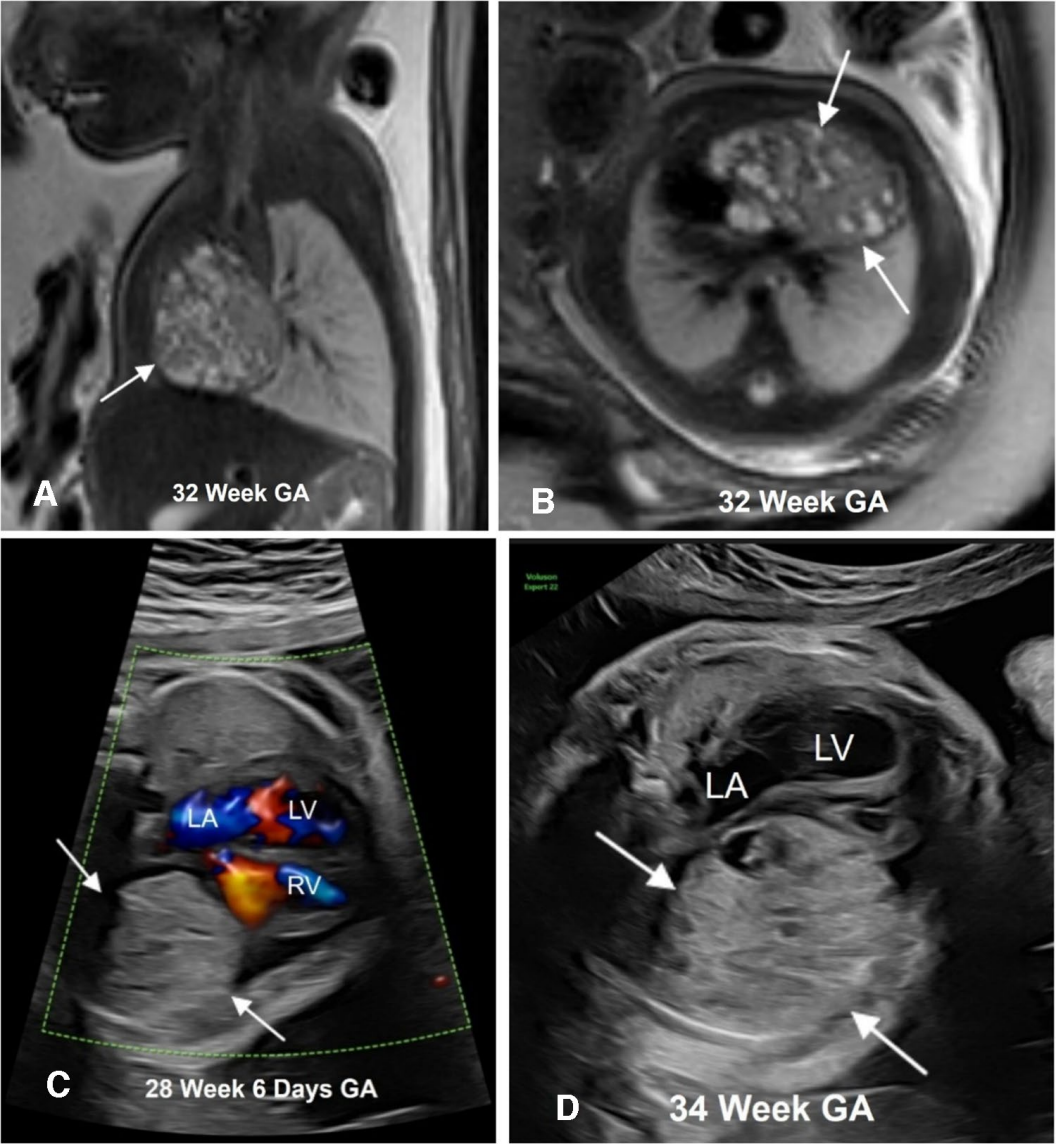
**A** Sagittal and (**B**) axial T2-weighted images from fetal MRI performed at 32 weeks gestational age demonstrating a complex cystic mass (*arrows*) centered in the right mediastinum with mass effect on the right atrium. **C** Axial horizontal view fetal echocardiogram at 28 weeks gestational age showing a heterogeneous echogenic mass (*arrows*) in the right chest adjacent to the right atrium and right ventricle. **D** Follow-up axial horizontal view fetal echocardiogram at 34 weeks gestational age shows increased size of the mass (*arrows*) with increased mass effect on the right atrium. The patient was delivered via C-section at 34 weeks 1 day due to the worsening hydrops and underwent immediate resection of the mass. Histopathology was consistent with congenital immature teratoma with no malignant components. *LA*, left atrium; *LV*, left ventricle; *RV*, right ventricle, *GA*, gestational age

Cardiac/pericardial teratoma is usually a well-defined, large, heterogeneous, T1 hypointense, and T2 hyperintense lesion. The complex cystic nature of the lesion can sometimes be better demonstrated on MRI than echocardiography. Intrinsic T1 hyperintensity utilized to detect intra-lesional fat or hemorrhage in postnatal teratoma is not well documented on fetal MRI, likely due to lower signal to noise of fetal T1 sequences and the relative lack of fat in a fetus. In our experience, none of the cardiac/pericardial teratomas demonstrated T1 hyperintensity on large field of view fast spoiled gradient echo (FSPGR) sequences. Larger lesions are typically centered in the middle mediastinum. Mass effect on the adjacent structures should be carefully evaluated, especially the atria and great vessels [34]. Figure 3 demonstrates echocardiography and fetal MRI appearance of a pericardial teratoma.

#### Treatment and outcomes

Due to associated large pericardial effusions, teratomas are often symptomatic early in life, if not prenatally [60]. Prenatal management consists of expectant management with close surveillance for tumors not causing hemodynamic compromise, with the knowledge that there can be rapid growth of the tumor. Postnatal surgical resection has a high rate of success. Generally, teratomas are benign and do not recur if resected completely. Favorable outcomes are less common in those with prenatal evidence of hydrops, with the liveborn percentage dropping from 95% to 58% [59]. If there is early evidence prenatally of declining cardiac output or hydrops, fetal interventions including pericardiocentesis, pericardio-amniotic or thoraco-amniotic shunt, or open fetal surgery have been successful in some cases [59, 60]. In patients closer to term with evidence of cardiac compromise or hydrops, pre-term delivery and ex utero intrapartum treatment may be considered, usually in conjunction with early resection.

### Fibroma

#### Epidemiology

Cardiac fibromas are the third most common fetal and second most common benign pediatric cardiac neoplasm [61]. They account for 5–12% of fetal cardiac tumors, but are overall rare with an estimated incidence of 1 in 280,000 births [4, 62]. There is an association with Gorlin-Goltz syndrome, with fibroma diagnosed in 3–5% of children affected with the syndrome [63]. Fibromas are typically solitary, located in the ventricular septum or the free wall of the left ventricle, and are larger than other fetal cardiac masses. Rarely, fibromas involve the right ventricular myocardium [64].

#### Echocardiographic features

Fibromas are typically solitary intra-myocardial lesions that are homogeneous and hypoechoic – often indistinguishable from rhabdomyomas by ultrasound. However, if cystic degeneration is present, they may be heterogenous or show calcification [32]. This often happens postnatally but has been described in fetal case reports [65]. Associated pericardial effusions have been reported, which are uncommon in rhabdomyomas and can be a distinguishing feature [65]. In addition to characterization of the mass, assessing for the presence of outflow tract obstruction and arrhythmias is essential.

#### MRI features

On fetal MRI, there are limited cases describing the MRI imaging of a fibroma. A case report of a prenatally confirmed fibroma with postnatal MRI performed at 4 days of age noted a solitary lesion with homogeneous T2 hypointense signal intensity and isointense T1 signal due to high cellularity and fibromyxoid stroma [66]. Prenatally, it cannot be confidently differentiated from a solitary rhabdomyoma, though decreased T2 signal may be helpful. Postnatally, intense late gadolinium enhancement in a cardiac fibroma is a differentiating feature. This is not possible to ascertain prenatally as gadolinium-based contrast agents are contraindicated in fetal imaging [34].

**Fig. 4:**
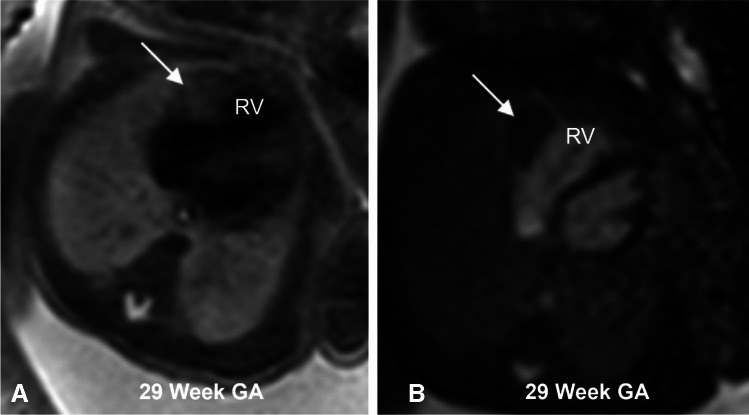
**A** Axial T2 and **B** balanced steady state free precession (bSSFP) images in a 29-week gestational age fetus demonstrating a well-defined mass (*arrows*) in the right ventricular wall, confirmed fibroma postnatally. *RV*, right ventricle; *GA*, gestational age

Fetal ocular anomalies including microphthalmia and vertebral segmentation anomalies have been reported in Gorlin-Goltz syndrome [26]. However, the known head and neck findings of odontogenic keratocysts, dural calcification, and medulloblastoma rarely have been reported prenatally. Figure 4 shows fetal MRI appearance of fibroma in a 29-week fetus.

#### Treatment and outcomes

The treatment approach for fetal cardiac fibromas depends on the size, location, and clinical impact. Unlike rhabdomyoma, cardiac fibromas do not regress and are more frequently associated with malignant ventricular arrhythmias [67]. Cardiac arrest and ventricular tachycardia are the most common clinical postnatal presentations, with cardiac arrest accounting for 24% and ventricular tachycardia for 55% in one case series [68]. Ventricular arrhythmias are a common indication for surgery, as surgical resection significantly reduces the risk of life-threatening ventricular arrhythmias. In patients where resection is not possible, arrhythmias are managed with anti-arrhythmic medications such as amiodarone or beta-blockers, and occasionally with implantable cardiac defibrillators [69]. Rarely, single ventricle palliation or cardiac transplant has also been used [69].

### Hemangioma

#### Epidemiology

Fetal cardiac hemangiomas are extremely rare with an unknown prenatal incidence. Postnatally, they account for 2–3% of primary cardiac tumors [70, 71]. Cardiac hemangiomas can be seen in isolation or in syndromes, such as visceral hemangiomatosis syndrome [72]. Case reports of syndrome-related fetal cardiac hemangiomas with multisystem involvement tend to have a higher incidence of fetal hydrops and Kasabach-Merritt phenomenon with subsequent poorer outcomes [73, 74].

Hemangiomas can arise anywhere near the heart, but the most common location is the base of the heart adjacent to the right atrium [4, 29, 72]. They are frequently associated with a pericardial effusion [75]. Similar to teratomas, careful monitoring is recommended to assess for worsening pericardial effusion, hemodynamic compromise, or the development of hydrops fetalis [76, 77].

#### Echocardiographic features

Hemangiomas are typically sessile, non-capsulated masses with mixed hyperechoic and hypoechoic areas [70]. The hypoechoic areas have been attributed to cavernous lakes and degeneration [78, 79] while the hyperechoic areas may be secondary to thrombus formation and calcification [80]. Although these are vascular tumors, hemangiomas typically do not typically demonstrate flow on color Doppler. Scant color signals may be seen around the periphery of the mass, but they are not as obviously vascularized as other extra-cardiac hemangiomas [81].

#### MRI features

**Fig. 5:**
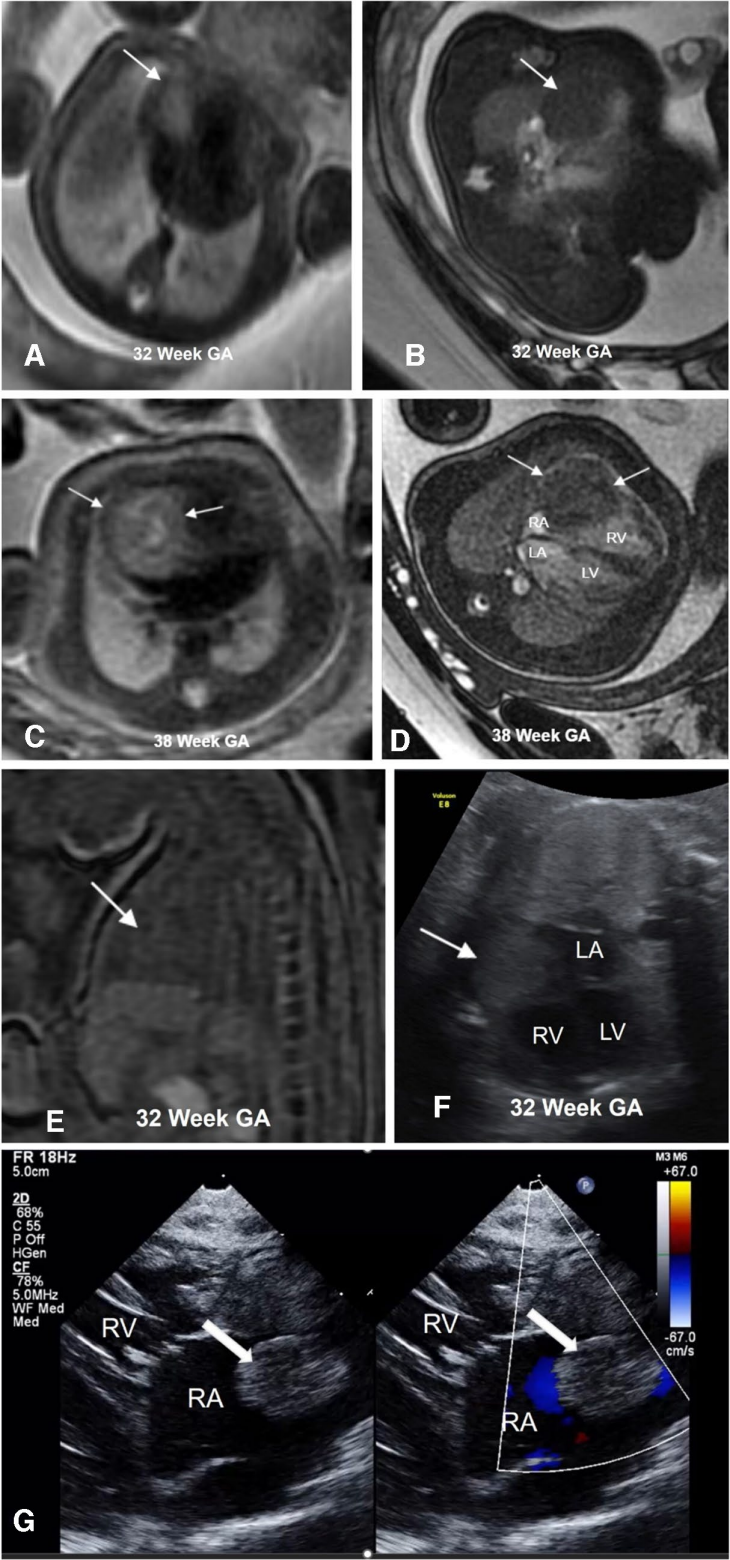
**A** Axial T2-weighted and (**B**) balanced steady state free precession images at 32 weeks gestational age showing a T2 hyperintense, balanced steady state free precession iso-hypointense mass (*arrow*) associated with the right atrium. Follow-up axial (**C**) T2 and (**D**) Balanced steady state free precession images at 38 weeks gestational age show increased size of the lesion (*arrows*). It is also more heterogeneous on T2-weighted images and is causing mass effect on the right atrium. **E** The sagittal fast spoiled gradient echo image does not demonstrate any intra-lesional hyperintensity to suggest fat or hemorrhage. **F** Axial 4-chamber view at 32 weeks gestational age demonstrates a homogeneous hyperechoic lesion (*arrow*) effacing the right atrium. **G** Postnatal parasternal long axis view echocardiogram angled toward the right ventricle at day of life 1 demonstrates the echogenic mass (*arrow*) with no appreciable intra-lesional flow on color Doppler on split screen view. The lesion was resected, and histopathology was consistent with a rapidly involuting congenital hemangioma. *RA*, right atrium; *LA*, left atrium; *RV*, right ventricle; *LV*, left ventricle; G*A*, gestational age

Baird et al. described a right atrial hemangioma on a neonatal MRI as a T2 hyperintense, enhancing mass with areas of intrinsic T1 shortening [82]. To the best of our knowledge, there are no published case reports describing the fetal MRI appearance of cardiac hemangioma. In our experience, the cardiac hemangioma described in Fig. 5 showed iso-hypointense signal on large field of view fast spoiled gradient echo (FSPGR) and balanced steady state free precession (bSSFP) sequences, and was T2 hyperintense relative to myocardium. It was initially homogeneous; however, on follow-up, it increased in size and became more heterogeneous with a central cystic component. The lesion distorted the right atrium, and there was a small associated pericardial effusion without hydrops. No additional fetal anomalies were seen.

#### Treatment and outcomes

With the rarity of these tumors, treatment remains controversial. Surgical resection is generally the preferred treatment, even in asymptomatic patients, due to the potential risk of embolism, rupture, and sudden death [72]. Medical therapies including steroids, beta-blockers, and radiotherapy have been reported [70, 72]. Hemangiomas associated with the atrial or ventricular septum have been associated postnatally with death [29, 72]. If complete resection is not possible, continued surveillance under the supervision of cardiologists and oncologists is recommended due to the potential for malignant transformation of the remaining mass.

### Pericardial cyst

**Fig. 6:**
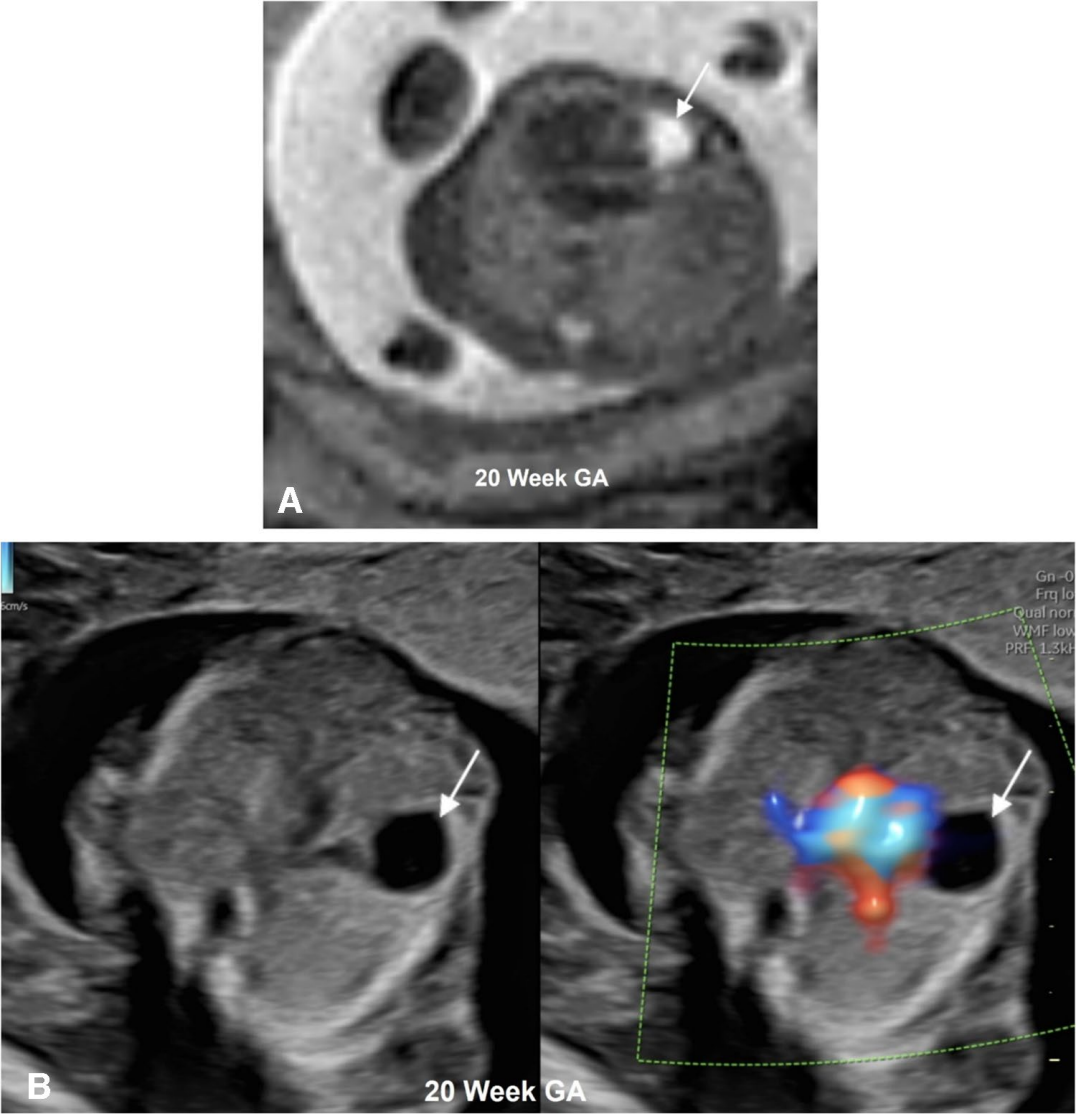
**A** Axial T2-weighted image in a 20-week gestation pregnancy showing a well-defined T2 hyperintense lesion (*arrow*) along the right cardiac border. **B** An axial fetal echocardiogram image in split screen view centered near the entrance of the inferior vena cava to the right atrium showing a well-defined anechoic cystic lesion (*arrow*) with no vascularity on color Doppler along the right cardiac border. *GA*, gestational age

Although not a true cardiac mass, pericardial cysts can rarely be confused for a cystic mass. Pericardial cysts are rare congenital mediastinal cysts with an incidence of ~1 per 100,000. The most common location is the right cardio-phrenic angle. On echocardiography, findings include an anechoic cystic lesion without any vascularity on color Doppler. On MRI, the cyst exhibits homogeneous T2 hyperintense signal. Differential considerations depend on the location and include bronchogenic cyst, enteric duplication cyst, and lymphatic malformation. These are usually asymptomatic and incidentally detected, and even larger lesions (3–4 cm) do not usually cause hemodynamic compromise [83]. Figure 6 shows the typical fetal imaging appearance of a pericardial cyst.

### Other benign lesions and mimics

Although extremely rare, other benign fetal cardiac masses have been reported in the literature. Myxomas, the most common benign cardiac tumors in adults, are rarely seen prenatally [9, 84–87]. In a review of 224 fetal and neonatal cardiac masses, Isacc et al. reported an incidence of 2.7% for myxomas [85]. In our personal experience, we have not encountered a confirmed case of prenatal cardiac myxoma. In the literature, these are described as echogenic pedunculated masses. Paladini et al. described a fetal cardiac myxoma which moved between the atrial chambers through the atrial septal defect, emphasizing the pedunculated nature as a distinct imaging feature [84]. Post natal resection is typically the preferred management due to the risk of embolization [54, 84].

**Fig. 7:**
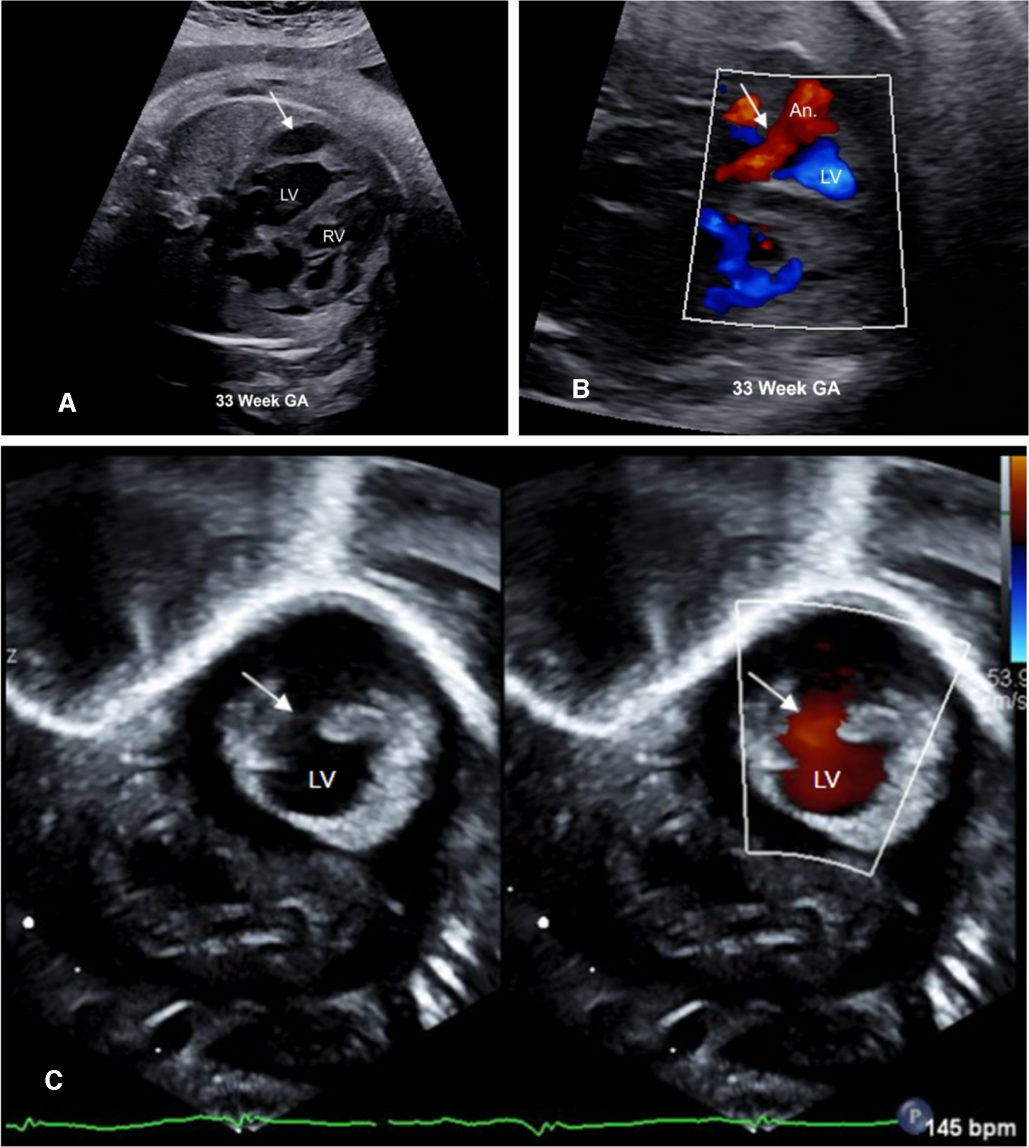
**A** Echocardiogram in a 33-week gestation fetus referred for evaluation of arrhythmia demonstrating hypoechoic lesion in the left ventricular wall (*arrow*). **B** Color Doppler image showing gap in the left ventricular wall (*arrow*) with flow into the aneurysm (*An*). **C** Postnatal echocardiography from a subcostal sagittal view confirmed the diagnosis of ventricular aneurysm. The LV to aneurysmal connection (*arrow*) is shown on split screen image. *LV*, left ventricle; *RV*, right ventricle; *An*, aneurysm

Cardiac lipomas have been described, with a case identified on fetal echocardiography as an echogenic lesion in the right ventricular outflow tract [9]. Right or left ventricular aneurysms or diverticula can also mimic cardiac masses, particularly if they are thrombosed [88]. Demonstration of communication with the cardiac cavity is helpful in the identification of an aneurysm or diverticula. A case of ventricular aneurysm is shown in Fig. 7. Zhou et al. also reported a case of massive pericardial effusion in which an enlarged thymic lobe indenting the pericardium and mimicked a teratoma [9].

## Conclusion

Most fetal cardiac masses are benign, with cardiac rhabdomyomas being the most common fetal cardiac mass. In the evaluation of fetal cardiac masses, echocardiography and MRI have a complementary role. The number and location of mass(es), morphological characteristics, and extra-cardiac findings such as the knowledge of any associated syndromes, are helpful clues in narrowing the differential diagnosis. Imaging features and diagnostic clues are summarized in Table 4. Multiple cardiac masses are pathognomonic of rhabdomyoma. A large solitary solid myocardial mass is usually a rhabdomyoma or fibroma. Teratomas are complex cystic lesions, typically pericardial in location and associated with pericardial effusion. Imaging plays a crucial role in the diagnosis, surveillance, and management of fetal cardiac masses. While most of these masses are benign, depending on their size and location, they can cause hemodynamic compromise requiring close pre- and postnatal surveillance.

**Table 4:** Summary of imaging features of each cardiac mass

Mass	Ultrasound	MRI signal compared to myocardium	Other diagnostic clues
Rhabdomyoma	Homogeneous echogenic lesions, often multiple	Homogeneous T1 iso- to hyperintense, T2 hyperintense	Multiple lesions, Inter-ventricular septum is most common location, genetically diagnosed tuberous sclerosis, extra-cardiac findings of tuberous sclerosis including subependymal nodules, cortical/subcortical tubers in brain and renal cysts,
Teratoma	Complex cystic lesion with calcification	Heterogeneous complex cystic mass T2 hyperintense relative to myocardium Can have T1 hyperintense areas reflecting intra-lesion fat or hemorrhage	Frequently para-cardiac rather than intra-cardiac; however, exact location may be difficult to assess due to mass effect on heart Pericardial effusion is more common
Fibroma	Maybe heterogeneous, can have calcification	T2 hypointense relative to myocardium, postnatally characteristic delayed mass-like enhancement pattern	Solitary, large lesion, Pericardial effusion is more common, associated with Gorlin-Goltz syndrome, arrhythmias are more common
Hemangioma	Echogenic well-defined lesion showing no or minimal internal vascularity on color doppler. Can have intra-lesional cystic spaces	T2 hyperintense, Iso to hyporintense on BTFE/bSSFP/cine sequences Postnatally, intense homogeneous enhancement	Most common location is along the right atrial wall Pericardial effusion is common

*bSSFP*, balanced steady state free precession; *BTFE*, balanced turbo free echo

## Data Availability

No datasets were generated or analysed during the current study.
